# Bradykinesia Is Driven by Cumulative Beta Power During Continuous Movement and Alleviated by Gabaergic Modulation in Parkinson's Disease

**DOI:** 10.3389/fneur.2019.01298

**Published:** 2019-12-20

**Authors:** Emma J. Prokic, Ian M. Stanford, Gavin L. Woodhall, Adrian C. Williams, Stephen D. Hall

**Affiliations:** ^1^School of Life and Health Sciences, Aston University, Birmingham, United Kingdom; ^2^Department of Biosciences, University of Helsinki, Helsinki, Finland; ^3^Queen Elizabeth Hospital, University Hospital Birmingham, Birmingham, United Kingdom; ^4^Brain Research and Imaging Centre, University of Plymouth, Plymouth, United Kingdom

**Keywords:** magnetoencephalography, oscillations, movement, GABA, zolpidem

## Abstract

Spontaneous and “event-related” motor cortex oscillations in the beta (15–30 Hz) frequency range are well-established phenomena. However, the precise functional significance of these features is uncertain. An understanding of the specific function is of importance for the treatment of Parkinson's disease (PD), where attenuation of augmented beta throughout the motor network coincides with functional improvement. Previous research using a discrete movement task identified normalization of elevated spontaneous beta and postmovement beta rebound following GABAergic modulation. Here, we explore the effects of the gamma-aminobutyric acid type A modulator, zolpidem, on beta power during the performance of serial movement in 17 (15M, 2F; mean age, 66 ± 6.3 years) PD patients, using a repeated-measures, double-blinded, randomized, placebo-control design. Motor symptoms were monitored before and after treatment, using time-based Unified Parkinson's Disease Rating Scale measurements and beta oscillations in primary motor cortex (M1) were measured during a serial-movement task, using magnetoencephalography. We demonstrate that a cumulative increase in M1 beta power during a 10-s tapping trial is reduced following zolpidem, but not placebo, which is accompanied by an improvement in movement speed and efficacy. This work provides a clear mechanism for the generation of abnormally elevated beta power in PD and demonstrates that perimovement beta accumulation drives the slowing, and impaired initiation, of movement. These findings further indicate a role for GABAergic modulation in bradykinesia in PD, which merits further exploration as a therapeutic target.

## Introduction

Spontaneous beta frequency (15–30 Hz) oscillations are a prominent electrophysiological signature of the primary motor cortex (M1) in humans (1, 2) and animal models (3). Beta oscillations have been posited as an “idling” rhythm of the motor system (4), in line with the conceptual need for a carrier signal to facilitate temporal binding of functional performance (5). From a functional perspective, beta power in M1 modulates in a task-dependent manner in relation to various phases of movement. Specifically, a bilateral reduction in beta is observed during movement preparation (6), with a further reduction in power at the onset of movement, referred to as movement-related beta desynchronization (MRBD) (7, 8). Beta power appears to be minimized during dynamic movement periods (9–11), where a reduction in beta power lasts as long as the total movement (4). Conversely, there appears to be an increase in beta power associated with static postural maintenance (12–14).

Following the completion or termination of movement, there is a transient increase in beta oscillatory power, which is elevated above the premovement baseline (1, 4, 8, 15), a phenomenon referred to as postmovement beta rebound (PMBR). With regard to functional significance, MRBD is a prerequisite for recruitment of functional assemblies ahead of movement (16), but is not dependent upon force, speed, direction (17), or movement time (18). The functional significance of PMBR is unclear, although it has been postulated as a marker of sensory reafference following movement (19) and appears to serve an inhibitory function (20). The interaction between MRBD and PMBR during the performance of serial movements is uncertain. However, it has been shown that beta activity is suppressed according to the likelihood of new motor processing, such that phasic suppression of beta activity before movement is replaced by a persistent suppression during a sequence of related movements, such as in finger tapping (FT) (21). In healthy humans, ability to effectively initiate movement is dependent upon an ability to achieve an absolute level of premovement desynchronization (22), and fast FT produces a persistent state of cortical beta desynchronization during movement (23).

In Parkinson's disease (PD), exaggerated beta oscillations are observed in recordings from subcortical structures, such as the subthalamic nucleus (STN) and cortex of both animal models (24–26) and PD patients (27, 28). There is some uncertainty regarding the importance of spontaneous cortical beta power in PD, as observations are typically in early-stage PD and show variation between participants (29), but an association between abnormal premovement desynchronization and deficits in movement initiation supports a role in akinetic symptoms in PD (30). Similarly, several studies report that this exaggerated beta power is attenuated following treatment with either levodopa (31, 32) or deep brain stimulation (33, 34), both of which are associated with relief of PD symptoms. We have previously reported that subsedative doses (2–5 mg) of the gamma-aminobutyric acid type A (GABA_A_) alpha-1 receptor modulator zolpidem improves cognitive and motor abilities of patients after stroke (35) and idiopathic PD (29), coincident with a reduction in beta power. This is consistent with previously observed GABA-mediated improvements in PD (36). In healthy controls, elevation of endogenous GABA levels is known to increase baseline beta power (37), as seen following administration of benzodiazepines (1, 2). From a functional perspective, the benzodiazepine Bromazepam, which has high alpha-2 and low alpha-1 subunit affinity for the GABA_A_ receptor, has been shown to have positive effects on motor learning involving focused attention in healthy participants (38).

Our previous research using magnetoencephalography (MEG) in PD, used the sedative hypnotic zolpidem to modulate activity at the gamma-aminobutyric acid type A (GABA-A) alpha-1 subunit. This demonstrated that PD patients exhibit impaired desynchronization in the movement preparation phase and increased amplitude and latency of the PMBR phase (29). These differences were reduced following administration of zolpidem and accompanied by improvement in symptomatic severity, measured by motor examination (Part III) of the Unified Parkinson's Disease Rating Scale (UPDRS). Given the evidence surrounding the potential for exaggerated beta in the motor system to impair effective movement in PD, via impediment of desynchronization, it is important to understand the potential role of perimovement beta power in this process. Here, we address this question through the investigation of oscillatory power, using MEG, in a serial movement task, using a simple FT paradigm. Following our previous study (29), we further explore the mechanisms of GABA-mediated functional improvements with low-dose zolpidem, in a cohort of PD patients presenting with unilateral symptoms.

We hypothesize that increased PMBR amplitude and latency in PD will produce a progressive accumulation of beta power over time, resulting in higher perimovement beta amplitude that impairs the ability to initiate subsequent movements. Furthermore, we postulate that greater perimovement beta power will coincide with an increased intertap interval (ITI). We predict that zolpidem will reduce perimovement beta power, affording an improvement in the performance of the finger-tapping speed and stability.

## Methods

### Participant Training and Assessment

We recruited 17 participants (15M, 2F), mean age of 66 ± 6.3 years, with a history of unilateral PD symptoms, following previous research (29). Consistent with ethical approval, patients continued with their prescribed medication during their participation in the experiment. Details of medication and other particulars were recorded for each participant (see Table 1 for details), although individual medication doses were not recorded. Drug and control experiments were conducted at the same time interval following medication to control for effects of other drugs. Five participants were eventually excluded from the analysis as bilateral impairments were observed at baseline. Participants attended the laboratory over 2 days, on which identical experimental protocols were used, with the exception of drug condition. A double-blinded and randomized approach was used to assign the order for each participant to either the drug-active (zolpidem) or placebo session. Before each neuroimaging experiment, each participant was trained in the motor task. Specifically, participants placed their hands on a magnetically silent acrylic plate, with the position of a flexible paddle beneath the index finger monitored using infrared light.

**Table 1:** Participant information summary.

**Patient ID**	**Gender**	**Dominant hand**	**Age (years)**	**Medication**	**Time since diagnosis (years)**	**Impaired side (L/R)**	**Δ UPDRS zolpidem^**#**^**	**Δ UPDRS Placebo^**#**^**
1	M	R	60	Ropinirole, Sinemet^a^	5	L	−12.80	2.07
2	F	R	67	Sinemet plus	1	L	−1.41	−4.54
3	M	R	67	Madopar^b^, Pramiprexole, Rasagline, Sinemet^a^	^*^	R	−12.79	3.03
4	M	R	72	Amantadine, Ropinorole CR, Sinemet CR^a^, Stalevo^c^	^*^	L	−12.05	−3.39
5	F	R	60	Co-careldopa^d^, Rasagiline	12	L	−1.17	9.62
6	M	R	67	Madopar^b^, Ropinirole, Selegeline	6	R	−7.44	4.06
7	M	R	82	Sinemet Plus^a^, Selegeline	5	R	−5.54	11.18
8	M	R	50	Ropinirole MR	^*^	L	−13.45	−5.25
9	M	R	72	Pramiprexole CR	3	R	−10.96	−12.54
10	M	R	69	Requip XL^e^, Stalevo^c^	6	R	−14.06	−12.63
11	M	R	57	Pramiprexole, Selegeline	^*^	R	−2.37	−8.48
12	M	R	67	Rasagiline, Sinemet^a^	4	L	−5.75	2.23

a *Carbidopa/L-dopa*,

b *benserazide/L-dopa*,

c *L- dopa/carbidopa/entacapone*,

d *carbidopa/L-dopa*,

e *ropinerole. MR, modified release; CR, controlled release*.

# *Mean change in time taken to complete each motor performance measurements*.

* *Time since diagnosis longer than 5-years, exact duration data were not available*.

### Motor Task and Symptom Assessment

As part of a functional task to localize M1, based upon PMBR (8, 15, 39), participants performed a visual reaction time task, in which they responded as quickly as possible to a change in visual cue with abduction of either the left or right index finger [e.g., (39)].

**Figure 1:**
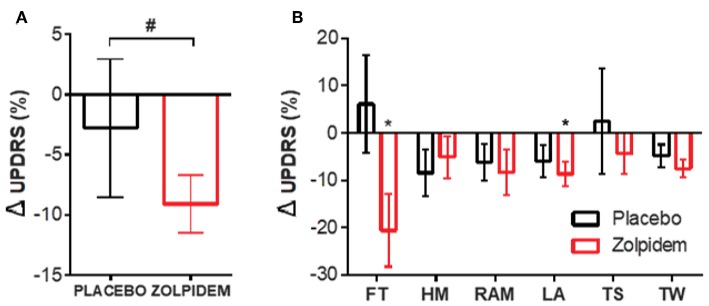
Motor performance measurements following placebo and zolpidem. **(A)** Total change (%) in motor performance score following placebo (black) and zolpidem (red). ^#^ INDICATES significant difference (*p* = 0.003) between conditions. **(B)** Motor performance, based upon Unified Parkinson's Disease Rating Scale (UPDRS) part III, was quantified (time to complete) and improvement between BL and drug condition calculated. Change (%) in scores are shown for placebo (black) and zolpidem (red) with significant change (^*^p < 0.05) indicated for finger tapping and leg agility (FT, finger tapping; HM, hand movement; RAM, rapid alternating movement; LA, leg agility; TS, time to stand; TW, time to walk).

The serial motor finger-tapping task consisted of six 10-s tapping trials, interleaved by 15-s periods of inactive rest. Participants were instructed to tap as quickly as possible following the onset of a “Start” cue, until the presentation of a “Stop” cue. Stimuli were presented on a projector screen, 1 m in front of the participant. Finger taps were recorded and analyzed, based upon triggers digitized from the infrared signals. The participant was instructed to relax their hand when the cue disappeared. Cue onset was jittered to minimize prediction effects. Participants rested their arm in a comfortable position, with the elbow and the lower arm resting on a flat surface. Before each MEG session, participants completed a series of tasks included in the UPDRS motor examination (Part III). This included FT, hand movement, rapid alternating movement, leg agility (LA), time to stand, and time to walk. Performance was quantified based upon the time taken to complete each task, rather than the typical 5-point scale, to increase sensitivity and reduce the variance of intrarater assessment (see Figure 1 for details). For each task, the rater used a stopwatch to record the time taken to complete a predetermined number of repetitions or distance.

### MEG and EMG Recordings

In each experiment, participants with normal or corrected to normal vision were seated in a 306-channel MEG system (Elekta, Finland). MEG data were acquired at a sampling rate of 1,000 Hz with a 50-Hz notch filter and 0.1–300 Hz bandpass filters. MEG data were coregistered with each participant's anatomical MRI, obtained using a 3-T MRI system (Siemens Magnetom Trio). This was achieved through surface matching of the MRI with a three-dimensional digitization of the participant's scalp (Fastrak, Polhemus, USA). Head position was monitored throughout, based upon the digitized position of five surface-mounted electromagnetic coils, positioned around the head. Electromyography (EMG), native to the MEG system, was used to record muscle activity from two disk electrodes placed upon the first dorsal interosseous muscle, simultaneously measured with the MEG acquisition.

At the end of the baseline (BL) recording session, participants were administered either oral zolpidem (0.05 mg/kg) or placebo, consistent with previously reported effective subsedative doses (35). An identical second MEG recording session was initiated 50 min after the zolpidem administration, with participants required to repeat the same rest and movement periods. Participants therefore completed a total of four MEG sessions (BL, zolpidem and BL, placebo).

### Data Analysis

Left and right M1 was localized using the synthetic aperture magnetometry beamforming method (40). Specifically, following finger movement, we identified the location of maximal contralateral beta power (15–30 Hz) increase in the PMBR period (500–1,000 ms postmovement termination) compared to rest (−2,000 to −1,500 ms premovement onset), following previous studies (8). Regions of interest identified from the beamforming analysis were used to determine the placement of virtual electrodes (40, 41), which were used to reconstruct neuronal network activity, specific to M1, over the envelope of the entire experiment. The power profile of the oscillatory activity was determined using Morlet wavelet time–frequency analysis of the virtual electrode output over the 1–100 Hz range in frequency bins of 0.5 Hz. For each participant, the individual beta peak was determined as the maximal peak of power spectral density in the 15–35 Hz range. This peak was then used to compute beta power changes in all subsequent analyses. Data from M1, contralateral to the affected hand, were grouped for further analysis and comparisons made between the baseline and drug (zolpidem) and control (placebo) conditions.

To determine the extent to which beta power increases during serial movement tasks (perimovement period), we used a cumulative summation (cusum) computation (Matlab R2019, Mathworks USA) to identify the progressive accumulation of power in the beta frequency range during the FT exercise. Specifically, the envelope of peak beta power during FT was reconstructed for each participant, in each condition. Data were converted to a zero-mean distribution, followed by sequential computation of the summed value of each sample over a 10-s period. We further explored whether a causal relationship exists between augmented beta and impaired sequential movement in PD. Peak beta power in the virtual electrode plots were used to reconstruct the profile of beta fluctuation associated with each tap during FT. The ITI and intertap variance (ITV) were computed as the mean and range, respectively, of the time between taps during the tapping task. Beta power was computed following each finger tap for each individual, for each condition. This was based upon the maximal beta amplitude in the interval between completion and initiation of movements, derived from the rectified EMG. Subsequently, we computed the number of taps in the average ITI following each beta peak to determine the relationship between beta amplitude and movement ability. All data are graphically represented as mean normalized change (%) ± SD. Groups were analyzed using two-way repeated measures ANOVA, with within-subject factors of “condition” (pre-/postdrug treatment) and “drug” (placebo/zolpidem). *Post-hoc t*-test comparisons are reported with Sidak's correction for comparisons. When only two groups were compared, a two-tailed paired *t*-test was used. No significant interactions were observed unless otherwise stated in the text.

## Results

### UPDRS Measures

The time-based UPDRS approach demonstrated a significant effect of time on the improvement in the symptomatic severity of participants [*F*_1,11_ = 12.00, *p* = 0.003]. *Post-hoc* analysis confirmed a significant improvement following administration of zolpidem [*t*_11_ = 3.34, *p* = 0.007] that was not seen following placebo [*t*_11_ = 1.557, *p* = 0.25] (Figure 1A). Further analysis of individual symptoms, using Sidak's multiple comparison test, confirmed a significant reduction [*t*_11_ = 2.36, *p* = 0.04; −1.59 ± 0.62 s] in the time taken to complete the FT task (20 taps) following zolpidem, which was not seen in the placebo condition [*t*_11_ = 0.19, *p* = 0.85; 0.28 ± 1.2 s] (Figure 1B, FT). Further analysis, using one-way repeated measures ANOVA, confirmed that there were no significant differences in tapping performance between the six tapping trials [*F*_(5,66)_ = 1.97, *p* = 0.12]; confirming that fatigue was not a driver of the observed changes. In addition, a similar improvement was observed in the LA task, where significant reduction in the time taken to complete 20 leg lifts following zolpidem [*t*_11_ = 2.39, *p* = 0.04; −0.72 ± 0.91 s] was not observed following placebo [*t*_11_ = 1.56, *p* = 0.24; −0.42 ± 0.80 s) (Figure 1B, LA).

### Reaction Time and Tapping Speed

**Figure 2:**
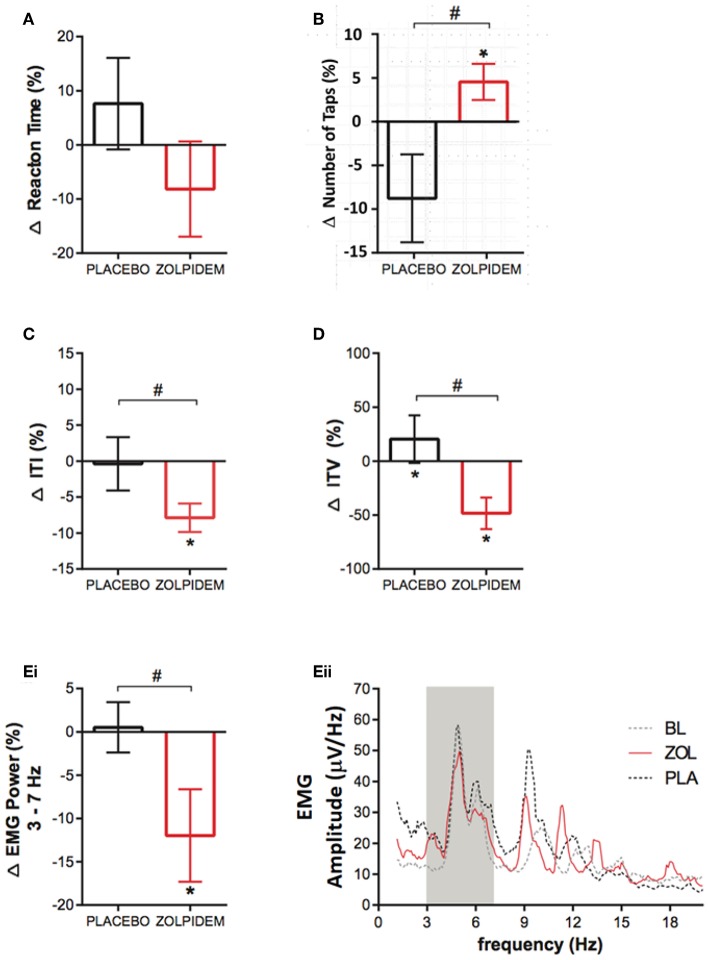
Functional measurements before and after placebo and zolpidem during magnetoencephalography (MEG) scanning. **(A)** Change (%) in latency of reaction time (RT) measured during the cued index-finger response task before and after placebo (black) and zolpidem (red). No significant effect of drug was observed (*p* = 0.78). **(B)** Mean change (%) in the number of taps completed in 10 s following placebo (black) and zolpidem (red) shows a significant difference (^#^) between conditions, as a result of an increase in the zolpidem condition (^*^p = 0.03). **(C)** Change (%) in the mean ITI during the completion of the 10-s tapping task following placebo (black) and zolpidem (red). Shows a significant difference (^#^) between conditions (*p* = 0.02). **(D)** Change (%) in the intertap variance (SD) following placebo (black) and zolpidem (red). Shows a significant difference (^#^) between conditions (*p* = 0.05). **(Ei)** Change (%) in EMG power in the 3–7 Hz frequency range following placebo (black) and zolpidem (red), which reveals a significant difference (^#^) following zolpidem (*p* = 0.04). **(Eii)** Shows the complete power spectral density profile of the EMG during baseline and after placebo and zolpidem. Gray box shows 3–7 Hz range.

Measurement of choice reaction time, using randomized left and right index finger movement cues, demonstrated no significant main effect of condition [*F*_1,11_ = 0.0003, *p* = 0.98] or drug [*F*_1,1_ = 0.09, *p* = 0.78] on the reaction time speed (placebo = 29.8 ± 31.8 ms; zolpidem = −28.5 ± 35.6 ms) (Figure 2A). Analysis of the FT task, completed in the MEG, was consistent with UPDRS findings (Figure 1B). A significant increase was observed in the number of taps completed following zolpidem [*t*_11_ = 2.61, *p* = 0.03; 1.21 ± 0.56 taps], which was not observed following placebo [*t*_11_ = 2.08, *p* = 0.11; 2.17 ± 1.34 taps) (Figure 2B). Consistent with an increase in tapping speed, a significant reduction in the ITI was observed following zolpidem [*t*_11_ = 2.85, *p* = 0.02; −26.68 ± 10.49 ms] but not placebo [*t*_11_ = 0.25, *p* = 0.96; 2.46 ± 5.10 ms) (Figure 2C). Furthermore, analysis of the ITV, which reflects the number of hastening and faltering events during the tapping task, revealed a significant reduction of ITV following zolpidem [*t*_11_ = 2.55, *p* = 0.05; −35.58 ± 17.38 ms] but not placebo [*t*_11_ = 0.29, *p* = 0.95; 15.08 ± 17.86 ms) (Figure 2D). Analysis of tremor amplitude, as measured by the 3–7 Hz frequency range power in the rectified EMG, revealed a significant main effect of drug [*F*_1,11_ = 9.49, *p* = 0.03], confirmed by *post-hoc* analysis as an amplitude reduction following zolpidem [*t*_11_ = 2.80, *p* = 0.04; −12.58 ± 16.04 μV/Hz] but not placebo [*t*_11_ = 0.05, *p* = 0.99; 1.52 ± 2.50 μV/Hz] (Figure 2Ei). This reduction is evident in the mean EMG power spectral density measures for each condition (Figure 2Eii).

### Beta Power and Movement Ability

**Figure 3:**
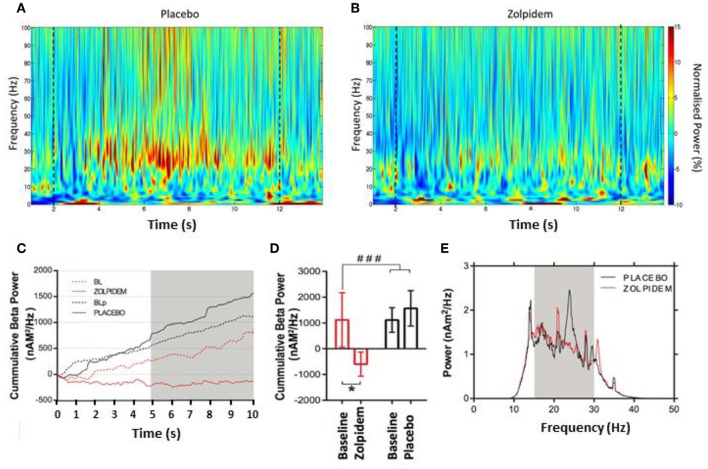
Beta power accumulation during the serial movement phase. Morlet–wavelet time–frequency spectrograms showing grand average power change, normalized to the pre-movement period for: **(A)** placebo and **(B)** zolpidem. Dashed lines indicate the start and end of movement in each condition. **(C)** Cumulative power (nAm^2^/Hz) in the beta (15–30 Hz) frequency bin during performance of the rapid tapping task. Graph demonstrates accumulation of beta power during baseline and placebo (dashed and solid back) and baseline and zolpidem (dashed and solid red), respectively. A clear increase in beta power is observed in both baseline and placebo conditions, but not following zolpidem, where suppression below baseline levels occurs. Gray box indicates the time interval where a significant difference (*p* < 0.05) can be seen between zolpidem and placebo conditions. **(D)** Mean cumulative beta (nAm^2^/Hz) in the baseline-and-placebo (black) and baseline-and-zolpidem (red) conditions. Significant difference (^*###*^*p* = 0.05) between drug conditions, with a significant difference (^*^) between zolpidem and baseline (*p* = 0.03), but not between placebo and baseline conditions (*p* = 0.52). **(E)** Power spectral density plot during the tapping period following placebo (black) and zolpidem (red). Significant reduction in the beta power to be centered around 25 Hz. Gray box shows the 15–30 Hz bin used to compute the power change.

Time–frequency analysis revealed an increase in beta power in the 10-s tapping period compared to the 2-s premovement baseline, which was more pronounced in the placebo than the zolpidem condition (Figures 3A,B). The accumulation of beta power over the 10-s period of FT, in each condition, was computed using the cumulative summation (cumsum) method (Matlab R2019, Mathworks USA). This revealed a significant main effect of drug [*F*_1,11_ = 3.96, *p* = 0.05), characterized by substantial accumulation of beta power in the baseline and placebo conditions but not in the zolpidem condition, where beta was suppressed below the premovement baseline (Figure 3C). *Post-hoc* analysis confirmed a significant reduction in the accumulation of beta power following zolpidem [−740 ± 548 nAm^2^/Hz; *t*_11_ = 2.39, *p* = 0.03] that was not observed following placebo [1,763 ± 1,389 nAm^2^/Hz; *t*_11_ = 0.66, *p* = 0.52) (Figure 3D). Frequency analysis, visualized as power spectral density estimation (Figure 3E) demonstrates that the reduction in cumulative beta power occurs with a peak at ~25 Hz, when compared to placebo. Further analysis of peak intertap beta, derived from the maximal beta amplitude in the interval between finger taps, revealed a significant main effect of drug [*F*_1,11_ = 7.28, *p* = 0.012] on intertap beta amplitude following zolpidem (−258.6 ± 123.5 nA/Hz: *t*_11_ = 3.91, *p* = 0.026] but not placebo [−0.85 ± 2.70%: *t*_11_ = 0.84, *p* = 0.11; 42.3 ± 126.8 nA/Hz].

**Figure 4:**
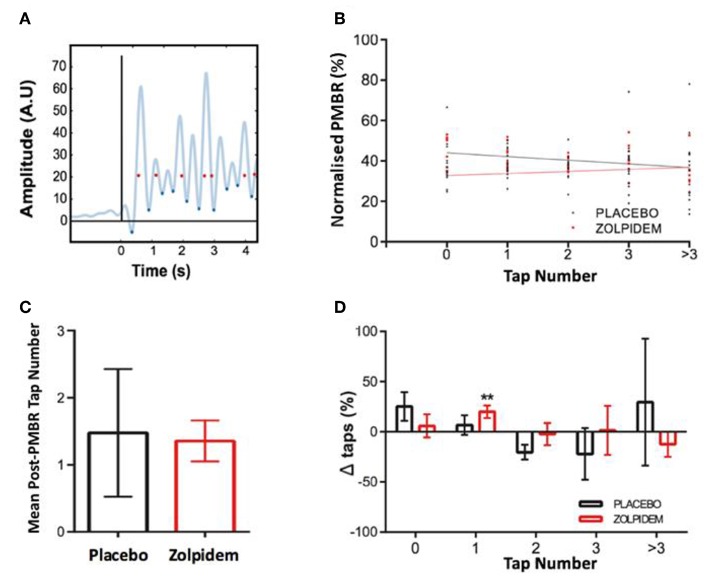
Perimovement beta power and movement execution. **(A)** Method for determining the relationship between high perimovement beta and movement execution. Trace shows rectified electromyography (EMG) trace (example from a single participant) from which the onset of movement was determined (blue diamonds) and temporally coregistered with the peak of each beta peak (red dots), determined from magnetoencephalography (MEG) virtual electrode (black line indicates task “Start” cue). **(B)** The amplitude peak beta (normalized as a percentage of the largest response) was computed following each event and assigned to the corresponding number of movements (taps) generated in the subsequent ITI. Mean amplitude of beta peaks and lines of best fit are shown for events in the placebo (black dots and line) and zolpidem (red dots and line) conditions. Plot shows the association between peak beta power and number of taps and significant reduction in the amplitude of beta in zero movement condition following zolpidem. **(C)** The mean number of taps per peak beta event (independent of power) is shown for the placebo (black) and zolpidem (red) conditions. There is no significant difference in the mean number of events but a notable reduction in the variance of the number of taps following zolpidem. **(D)** The change in the composition of missed, single, and multiple taps in the placebo (black) and zolpidem (red) condition. A significant increase (^**^) in the number of single taps (*p* = 0.008).

Subsequent analysis of the causal relationship between perimovement beta power and movement ability was performed by computing the number of finger taps, derived from the rectified EMG, occurring within each participant's mean ITI following each intertap beta peak (Figure 4A). In the baseline and placebo conditions, an inverse correlation was observed between the number of taps performed and beta amplitude (*R*^2^ = 0.96, *p* = 0.003). Following administration of zolpidem, a substantial reduction in beta power was observed, with greatest reduction associated with the absence of taps (*R*^2^ = 0.58, *p* = 0.14, Figure 4B).

Power-independent analysis of the number of taps/peak-beta event revealed no significant change [*t*_11_ = 1.21, *p* = 0.28] in the mean number of taps following each peak between placebo (1.48 ± 0.95) and zolpidem (1.36 ± 0.31) conditions. However, a substantial change in the variance was observed, indicating a reduction in the number of zero and multiple taps (Figure 4C). Analysis of the individual beta peak events revealed a significant main effect of drug on the number of single taps [*F*_1,11_ = 6.02, *p* = 0.03]. Sidak corrected *post-hoc* comparison confirmed a significant increase in the number of single taps following zolpidem [20.04 ± 1.52%; *t*_11_ = 3.36, *p* = 0.0079], but not following placebo [6.78 ± 9.83%: *t*_11_ = 0.6, *p* = 0.62]. This is likely to be accounted for by modest and non-significant changes to the number of missed and multiple taps (Figure 4D).

## Discussion

This study expands upon previous observations that PMBR is elevated in PD patients to demonstrate a progressive accumulation of beta power during the course of a serial tapping task. Further analysis shows that the ability to generate individual taps is directly associated with beta power in the preceding interval. Improvement in motor performance, observed following administration of zolpidem, is associated with a reduction in the accumulation of perimovement beta power.

### Perimovement Beta as an Inhibitory Signal in PD

These experiments demonstrate an important mechanism by which abnormally elevated beta power following movement may impair the ability of patients to perform subsequent and therefore serial movements in PD. As demonstrated in previous experiments, there appears to be an augmentation of beta oscillatory power throughout the motor network of PD patients (27, 28, 42), which appears to be reduced following effective therapeutic treatment that improves symptomatic presentation (31–33). Importantly, however, although numerous studies imply a connection between the beta signal and movement impairments in PD, the causal association is at present inconclusive (43, 44). However, the changes observed in these cases are typically modest, offering an inconclusive explanation for augmented spontaneous beta power as a mechanism for inhibition of movement. A common limitation of laboratory studies exploring motor function is the discrete nature of the tasks, in which participants are typically required to perform individual movements separated by several seconds. Given that the majority of movements and the impairments that arise PD are serial in nature and form part of a sequence of repeated or interconnected actions, it is unsurprising that studies of discrete individual movements are unable to offer adequate explanation for the effect of augmented beta power on movement. Specifically, while mean spontaneous beta power may be augmented, it is by no means continuous and tends to manifest as “bursts” of elevated beta power at rest (29, 45), which is possibly a consequence of the temporal fluctuations in endogenous dopamine (DA) release (43). A causal association between elevated beta and impaired movement could be implied by an increased statistical probability of impaired movement associated with the burst period, a concept that is supported by the observed success of adaptive deep brain stimulation, whereby stimulation is applied in response to elevated beta power (46). Recent studies in the STN demonstrate that beta bursts persist during movement and coincide with reduced velocity, consistent with bradykinesia and with mechanistic theories of adaptive deep brain stimulation (47, 48). This is in contrast to a positive correlation between gamma burst amplitude and velocity (49). Moreover, stimulation of M1 in control participants using tACS shows that stimulation at beta frequency reduces motion amplitude during a repetitive movement paradigm (50).

Here, we demonstrate the inhibitory nature of a functionally related neuronal network feature, perimovement beta. This observation is consistent with the exaggerated PMBR that we have previously shown to be abnormally elevated and sustained in PD (29). PMBR, an inhibitory signature, is unavoidably generated following movement and, therefore, when elevated and sustained in PD, has a greatly increased probability of impairing subsequent movements. In the present study, we demonstrate the impact of an accumulation of beta power (Figure 3C) and propose this as a critical mechanism in the inhibition of continuous movement in PD. We further demonstrate the relationship between the amplitude of individual beta-peak events and ability of patients to initiate subsequent movements. The observation of cumulative cortical beta power during sequential FT is an important addition to our understanding, as healthy controls exhibit persistent beta suppression in the motor cortex during continuous movement (23), and this is also seen in the STN of PD patients (21).

### GABAergic Improvement of Serial Movement

There is substantial evidence in support of the role of DA dysfunction underlying motor symptoms in PD (51). In particular, a decline in dopaminergic nigrostriatal projections in the basal ganglia (BG) resulting in reduction in excitatory drive to the direct pathway and inhibitory drive to the indirect pathway (52, 53). However, while DA undoubtedly plays a critical role in regulating the activity of cortico-BG-thalamic circuit, the predominant connections within this system are GABAergic and glutamatergic. Within the BG, GABAergic projections are the predominant connection between the striatum and globus pallidus [pars interna (GPi) and pars externa (GPe)], GPe to GPi, GPi to STN, GPi to thalamus, and GPi to brainstem [see (54) for a summary]. In addition, activity in the M1 and primary somatosensory cortex (S1) is GABAergically mediated (1, 8, 55, 56). It is, therefore, unsurprising that administration of a specific GABA-A alpha-1 modulator such as zolpidem elicits a change in motor function in PD.

These findings raise several important questions on the mechanistic nature of elevated PMBR and GABA-mediated desynchronization and improvement in PD. Previous studies have demonstrated that M1 beta power is driven by GABAergic interneuron-mediated synchrony, which is contingent upon excitatory inputs (56). Prevailing PD theories suggest that an increase in inhibitory GP to thalamic drive reduces excitatory input to the cortex (57), which suggests that PMBR does not occur in response to thalamo-cortical inputs. One might speculate that thalamo-cortical inputs to a putative M1 layer 4 (58) may be temporally aligned to postmovement sensory feedback from S1. This would present a mechanism for motor efficiency, whereby sensory information elicits direct influence over motor feedback from the cortico-BG-thalamocortical loop, providing an opportunity for optimization through plastic change. A potential consequence of such integration is the attenuation of the strength of feedback from S1 to M1, primarily in layers II/III (59, 60). Given the influence of S1–M1 connectivity on oscillatory power in the beta frequency range (55), this presents an appealing hypothesis for PMBR function and abnormal attenuation in PD. This suggestion is consistent with that of afferent feedback and sensorimotor recalibration following a period of change (18).

The mechanism by which GABAergic modulation attenuates abnormally elevated PMBR, as previously reported (29), is uncertain. Previous studies observe that GABA-A modulators augment spontaneous beta power in the motor cortex, through increased drive to local interneurons in healthy control participants (1, 2, 55, 56). Further observations in healthy controls, that PMBR is unaffected by GABA-A modulation (8), raises the possibility that a separate subcortical mechanism, involving GABAergic projections in the BG, is a plausible site of action for the effects observed here. An alternative cortical mechanism, specific to the low-dose administration of zolpidem, has previously been described (61), in which low-dose zolpidem selectively augments interneuron (fast spiking) specific GABA-A mediated tonic currents, resulting in reduction in beta oscillatory power. Regardless of the precise mechanism by which these changes occur, these findings reiterate the relatively untapped potential for engagement with GABAergic projections throughout the motor system, as a target for therapeutic development in PD. The observed zolpidem-specific improvements and associated oscillatory changes in the present study raises further questions about the potential impact of low-dose modulation in non PD participants. While the results of previous research (29) shows that discrete movements and associated oscillatory changes are unchanged in healthy participants, the addition of an age-matched control group would serve to clarify the current findings further. In conclusion, these findings provide consistent evidence for the role of beta oscillations in the symptomatic presentation in PD. In particular, we demonstrate a mechanistic process whereby cumulative beta, generated during repeated movement, is disruptive to the generation of serial motor output. Moreover, we demonstrate the involvement of GABAergic units in the generation of beta hypersynchrony, which can be attenuated through modulation of GABA-A alpha-1 receptor activity.

## Data Availability Statement

The datasets generated for this study are available on request to the corresponding author.

## Ethics Statement

The studies involving human participants were reviewed and approved by School of Life and Health Sciences Ethics Committee, Aston University. The patients/participants provided their written informed consent to participate in this study.

## Author Contributions

EP: experimental design, data collection, analysis, and manuscript preparation. GW, IS, and SH: experimental design, analysis, and manuscript preparation. AW: experimental design, PD assessment support, and manuscript preparation.

### Conflict of Interest

The authors declare that the research was conducted in the absence of any commercial or financial relationships that could be construed as a potential conflict of interest.
